# Antibacterial Activity of a Novel Peptide-Modified Lysin Against *Acinetobacter baumannii* and *Pseudomonas aeruginosa*

**DOI:** 10.3389/fmicb.2015.01471

**Published:** 2015-12-22

**Authors:** Hang Yang, Mengyue Wang, Junping Yu, Hongping Wei

**Affiliations:** Key Laboratory of Special Pathogens and Biosafety, Center for Emerging Infectious Diseases, Wuhan Institute of Virology, Chinese Academy of SciencesWuhan, China

**Keywords:** bacteriophage lysin, engineered lysin, *Acinetobacter baumannii*, *Pseudomonas aeruginosa*, outer membrane permeabilizers (OMPs)

## Abstract

The global emergence of multidrug-resistant (MDR) bacteria is a growing threat to public health worldwide. Natural bacteriophage lysins are promising alternatives in the treatment of infections caused by Gram-positive pathogens, but not Gram-negative ones, like *Acinetobacter baumannii* and *Pseudomonas aeruginosa*, due to the barriers posed by their outer membranes. Recently, modifying a natural lysin with an antimicrobial peptide was found able to break the barriers, and to kill Gram-negative pathogens. Herein, a new peptide-modified lysin (PlyA) was constructed by fusing the cecropin A peptide residues 1–8 (KWKLFKKI) with the OBPgp279 lysin and its antibacterial activity was studied. PlyA showed good and broad antibacterial activities against logarithmic phase *A. baumannii* and *P. aeruginosa*, but much reduced activities against the cells in stationary phase. Addition of outer membrane permeabilizers (EDTA and citric acid) could enhance the antibacterial activity of PlyA against stationary phase cells. Finally, no antibacterial activity of PlyA could be observed in some bio-matrices, such as culture media, milk, and sera. In conclusion, we reported here a novel peptide-modified lysin with significant antibacterial activity against both logarithmic (without OMPs) and stationary phase (with OMPs) *A. baumannii* and *P. aeruginosa* cells in buffer, but further optimization is needed to achieve broad activity in diverse bio-matrices.

## Introduction

The global emergence of multidrug-resistant (MDR) Gram-negative bacteria is a growing threat to public health worldwide (Li et al., 2015). Due to the diversity of resistance mechanisms that may lead to MDR or even pandrug resistance (PDR; Livermore and Woodford, 2006; Potron et al., 2015), *Acinetobacter baumannii* and *Pseudomonas aeruginosa* are among the increasingly reported and commonly identified MDR or even PDR nosocomial pathogens. They are responsible for many hospital-acquired infections, ranging from mild skin wounds and urinary tract infections to severe life-threatening infections, including bloodstream, pneumonia, and meningitis (Garcia-Quintanilla et al., 2013; Bassetti et al., 2014). At the same time, the progress in developing new antibiotics against these pathogens is slow (Fischbach and Walsh, 2009). Therefore, new strategies for controlling these MDR pathogens are urgently needed.

Bacteriophage lysins, the weapon of phages to digest the host bacterial cell wall for the release of progeny phages, have been extensively demonstrated to be promising alternatives in the treatment of Gram-positive pathogens, such as staphylococci and streptococci (Nelson et al., 2012; Pastagia et al., 2013; Yang et al., 2014). Due to their unique working mechanisms, lysins possess a low possibility of developing resistance (Fischetti, 2008; Knoll and Mylonakis, 2014). However, the outer membranes of Gram-negative bacteria block the access of natural lysins to their peptidoglycan substrates, thus making the exogenously added lysins useless or very weak against the viability of the target cells (Morita et al., 2001; Lai et al., 2011; Lim et al., 2014).

Currently, several strategies have been developed to break the barriers posed by the outer membranes of Gram-negative bacteria to natural lysins. Physical (i.e., high hydrostatic pressure; Briers et al., 2008) and chemical permeabilizers (i.e., EDTA, and weak organic acid, usually citric acid; Briers et al., 2007, 2011) can permeabilize the outer membrane efficiently to enhance the antibacterial activity of lysins, but are only applicable in applications such as food conservation and the treatment of topical infections. Structure-based engineering and phage genome-based screening methods have also been used to find novel lysins that act on *Yersinia* with the FyuA receptor (Lukacik et al., 2012) and *A. baumannii* (Lood et al., 2015). In recent years, a few engineered lysins have been reported with good antibacterial activities against *P. aeruginosa* by fusing natural lysins with optimized N- or C-terminal lipopolysaccharides-destabilizing or antimicrobial peptides, respectively, which can permeabilize the outer membranes (Briers et al., 2014a,b). In principle, considering the easy availability of lipopolysaccharide-destabilizing and antimicrobial peptides, fusing natural lysins with such peptides looks quite attractive since it provides a good way with plenty of chances to create novel engineered lysins against Gram-negative bacteria. However, reports on peptide-modified lysins are limited, and mainly focus on *P. aeruginosa*.

In the present study, a new peptide-modified lysin (PlyA) against *A. baumannii* and *P. aeruginosa* was constructed by fusing the cecropin A peptide residues 1–8 (KWKLFKKI) with the OBPgp279 lysin (Walmagh et al., 2012), and its antibacterial activity was evaluated.

## Materials and Methods

### Bacterial Strains

All bacterial strains and clinical isolates (**Table 1**) used in this work were grown in Luria Broth (LB) medium at 37°C. All clinical isolates of *A. baumannii* and *P. aeruginosa* were identified by 16S rDNA sequencing analysis combined with the biochemistry test using a MicroStation system (Biolog, GEN III Omnilog Combo Plus System, USA).

**Table 1:** Bacterial strains and clinical isolates used in this work.

Strain	Antibiogram	Strain	Antibiogram
***Acinetobacter baumannii*^a^**		***Pseudomonas aeruginosa*^a^**	
WHG3047	ND	WHG3012	Ak^S^, Ge^S^, Cf^S^, Im^S^
WHG3051	ND	WHG3022	Ak^S^, Ge^R^, Cf^R^, Im^R^
WHG3059	ND	WHG3029	Ak^S^, Ge^S^, Cf^S^, Im^S^
WHG3072	Ak^R^, Ge^R^, Cf^R^, Im^R^	WHG3014	Ak^S^, Ge^S^, Cf^S^, Im^S^
WHG3074	Ak^R^, Ge^R^, Cf^R^, Im^R^	WHG3015	Ak^S^, Ge^S^, Cf^R^, Im^S^
WHG3075	Ak^R^, Cf^R^, Im^R^	WHG3028	Ak^S^, Ge^S^, Cf^R^, Im^R^
WHG3032	Ak^S^, Ge^S^, Cf^S^, Im^S^	WHG3030	Ak^S^, Cf^S^, Im^S^
WHG3064	Ak^S^, Ge^S^, Cf^S^, Im^S^	WHG3013	Ak^R^, Ge^R^, Cf^R^, Im^R^
WHG3078	Ak^R^, Ge^R^, Cf^R^, Im^R^	WHG3043	Ak^S^, Ge^S^, Cf^S^, Im^R^
WHG3048	ND	WHG3006	Ak^S^, Ge^S^, Cf^S^, Im^S^
WHG3049	ND	WHG3007	Ak^S^, Ge^S^, Cf^S^, Im^R^
WHG3050	ND	WHG3008	Ak^S^, Ge^S^, Cf^S^, Im^R^
WHG3052	ND	WHG3009	Ak^S^, Ge^S^, Cf^S^, Im^R^
WHG3053	ND	WHG3016	Ak^S^, Ge^S^, Cf^S^, Im^I^
WHG3054	ND	WHG3018	Ak^S^, Ge^S^, Cf^S^, Im^S^
WHG3082	Ak^S^, Ge^S^, Cf^S^, Im^S^	WHG3021	Ak^S^, Ge^S^, Cf^S^, Im^R^
WHG3073	Ak^R^, Ge^R^, Cf^R^, Im^R^	WHG3019	Ak^S^, Ge^S^, Cf^R^, Im^R^
WHG3070	Ak^R^, Ge^R^, Cf^R^, Im^R^	WHG3024	Ak^R^, Ge^R^, Cf^R^, Im^R^
WHG3071	Ak^R^, Ge^R^, Cf^R^, Im^R^	WHG3025	Ak^S^, Ge^S^, Cf^S^, Im^R^
WHG3077	Ak^R^, Ge^R^, Cf^R^, Im^R^	WHG3026	Ak^S^, Ge^S^, Cf^S^, Im^S^
WHG3083	Ak^R^, Ge^R^, Cf^R^, Im^R^	WHG3031	Ak^R^, Ge^R^, Cf^R^, Im^R^
WHG3079	Ak^R^, Ge^R^, Cf^R^, Im^R^	WHG3033	Ak^S^, Ge^S^, Cf^S^, Im^R^
WHG3080	Ak^R^, Ge^R^, Cf^R^, Im^R^	WHG3034	Ak^S^, Ge^S^, Cf^S^, Im^S^
WHG3081	Ak^R^, Ge^R^, Cf^R^, Im^R^	WHG3036	Ak^S^, Ge^S^, Cf^S^, Im^S^
WHG3056	Ak^S^, Ge^S^, Cf^S^, Im^S^	WHG3017	Ak^S^, Ge^S^, Cf^S^, Im^S^
WHG3058	Ak^R^, Ge^R^, Cf^R^, Im^R^	WHG3037	Ak^S^, Ge^S^, Cf^S^, Im^S^
WHG3061	Ak^R^, Ge^R^, Cf^R^, Im^R^	WHG3039	Ak^S^, Ge^R^, Cf^S^, Im^S^
WHG3063	Ak^S^, Ge^R^, Cf^R^, Im^R^	WHG3038	Ak^R^, Ge^R^, Cf^R^, Im^R^
WHG3062	Ak^R^, Ge^R^, Cf^R^, Im^S^	WHG3040	Ak^S^, Ge^S^, Cf^I^, Im^S^
WHG3065	Ak^R^, Ge^R^, Cf^R^, Im^R^	WHG3041	Ak^S^, Ge^S^, Cf^S^, Im^S^
WHG3068	Ak^S^, Ge^R^, Cf^R^, Im^R^	WHG3042	Ak^S^, Ge^S^, Cf^S^, Im^S^
WHG3066	Ak^S^, Ge^S^, Cf^R^, Im^R^	WHG3023	Ak^S^, Ge^S^, Cf^S^, Im^S^
***Escherichia coli^b^***		WHG3027	Ak^S^, Ge^S^, Cf^R^, Im^R^
BL21(DE3)	ND	WHG3029	Ak^S^, Ge^S^, Cf^S^, Im^S^
***Bacillus cereus^b^***		WHG3014	Ak^S^, Ge^S^, Cf^S^, Im^S^
IS195	ND	WHG3015	Ak^R^, Ge^R^, Cf^S^, Im^R^

*Antibiogram: Ak, amikacin; Ge, gentamicin; Cf, cefepime; Im, imipenem; ND, not detected; R, resistant; S, susceptible; I, intermediate-resistant*.

*^a^Isolated from Zhongnan Hospital of Wuhan University; ^b^Lab collection*.

### Construction of Plasmids

Cecropin A is a 37-residue membrane-active antimicrobial polypeptide that kills bacteria by dissipating transmembrane electrochemical ion-gradients (Silvestro and Axelsen, 2000). Because the N-terminal residues 1–8 of cecropin A (CA, KWKLFKKI) are highly positively charged, this fragment was used in this study. OBPgp279 coding sequence was initially cloned into the modified pET28a(+) plasmid (Kan^R^) containing a (G4S)_2_ liner between *Bam*HI and *Eco*RI sites (Yang et al., 2012), using primers OBP-F (5-tatagaattcatgaaaaactcggaaaagaacg-3) and OBP-R (5-atatctcgagcacgatacccagagcttttttg-3), to obtain pET-*OBPgp279* vector (Kan^R^). The coding sequence for cecropin A peptide residues 1–8 (CA, KWKLFKKI) was cloned into the pET-*OBPgp279* vector, using primers CA-F (5-catgggcaaatggaaattatttaagaaaattg-3) and CA-R (5-gatccaattttcttaaataatttccatttgcc-3), to obtain pET-CA-*OBPgp279* (pET-PlyA, Kan^R^) vector. After verification by sequencing, *E. coli* BL21(DE3) cells were transformed with the correct plasmid for protein expression.

### Protein Purification

The recombinant enzymes were purified as described previously in our laboratory (Huang et al., 2015). Briefly, the *E. coli* BL21(DE3) cells were induced with 0.5 mM isopropyl β-D-thiogalactoside (IPTG) overnight at 16°C and collected for protein purification after sonication on ice. Then the proteins were collected by washing and eluting with 80 and 400 mM imidazole through a nickel nitrilotriacetic acid column (GE Healthcare, US), respectively. The collected active protein fractions were pooled and dialyzed against 20 mM Tris-HCl (pH 7.4) and stored at –80°C until use (less than 2 weeks).

### Antibacterial Activity Assay

To determine the antibacterial activity of PlyA, logarithmic (cultured for 3–4 h, OD_600_ = 0.6–0.8) or stationary phase (cultured for 14–16 h, OD_600_ = 1.4–1.6) cultures of *A. baumannii* WHG3066 were centrifuged (10,000 *g* × 1 min) first. Then the cells were washed once and resuspended in 20 mM Tris-HCl (pH 7.4). Bacterial suspensions (100 μl) were mixed with the enzyme in the presence or absence of the outer membrane permeabilizers (OMPs, i.e., EDTA and citric acid) at 37°C for 15–60 min. Finally, the remaining viable cells were calculated by plating onto LA plates. For susceptibility test, clinical isolates of *A. baumannii* and *P. aeruginosa* were cultured to logarithmic phase and treated with 50 (for *A. baumannii* isolates) or 100 μg/ml (for *P. aeruginosa* isolates) PlyA at 37°C for 1 h. All assays were performed for at least three times in biological repeats.

To test the synergism between OMPs and PlyA, stationary phase *A. baumannii* WHG3066 and *P. aeruginosa* WHG3012 cells were treated with 100 μg/ml PlyA in the presence of various concentrations of EDTA or citric acid in 20 mM Tris-HCl (pH 7.4) or 5 mM HEPES-NaOH (pH 7.4) at 37°C for 1 h. Afterward, the viable cell numbers were counted by plating. To avoid the acidification effect of citric acid, the synergism between citric acid and PlyA was also performed by adjusting the pH values of the reaction systems to 7.4. All assays were performed for at least three times in biological repeats.

To test the effects of bio-matrix on the antibacterial activity of PlyA, *A. baumannii* WHG3066 cells in logarithmic phase were washed once with 20 mM Tris-HCl buffer (pH 7.4), and resuspended in medium, including LB, Mueller–Hinton (MH, Huankai Microbial, Guangdong, China), Brain Heart Infusion (BHI, Huankai Microbial, Guangdong, China) and Tryptic Soy Broth (TSB, Becton, Dickinson & Co., France) with 4% NaCl (TSBN), or pasteurized milk (Mengniu Group, Wuhan, China), or human serum (Sigma–Aldrich, Shanghai, China). Then, cells were treated with 100 μg/ml PlyA at 37°C for 1 h, respectively. The viable cell numbers were evaluated by plating on LA plates. All assays were performed for at least three times in biological repeats.

### Transmission Electron Microscope (TEM)

The action of PlyA on the cell wall of the bacteria was monitored by a thin-section transmission electron microscope (Tecnai G^2^ 20 TWIN, FEI, USA). Briefly, *A. baumannii* WHG3066 cell suspensions in logarithmic phase were incubated with 100 μg/ml PlyA at 37°C for 10, 15, and 30 min, respectively. Then, the reactions were terminated by addition of 2.5% glutaraldehyde and the fixed samples were analyzed by transmission electron microscope (TEM). Cells treated with 20 mM Tris-HCl (pH 7.4) under the same conditions were used as controls.

## Results

### Characteristics of PlyA in Tris-HCl Buffer

**FIGURE 1:**
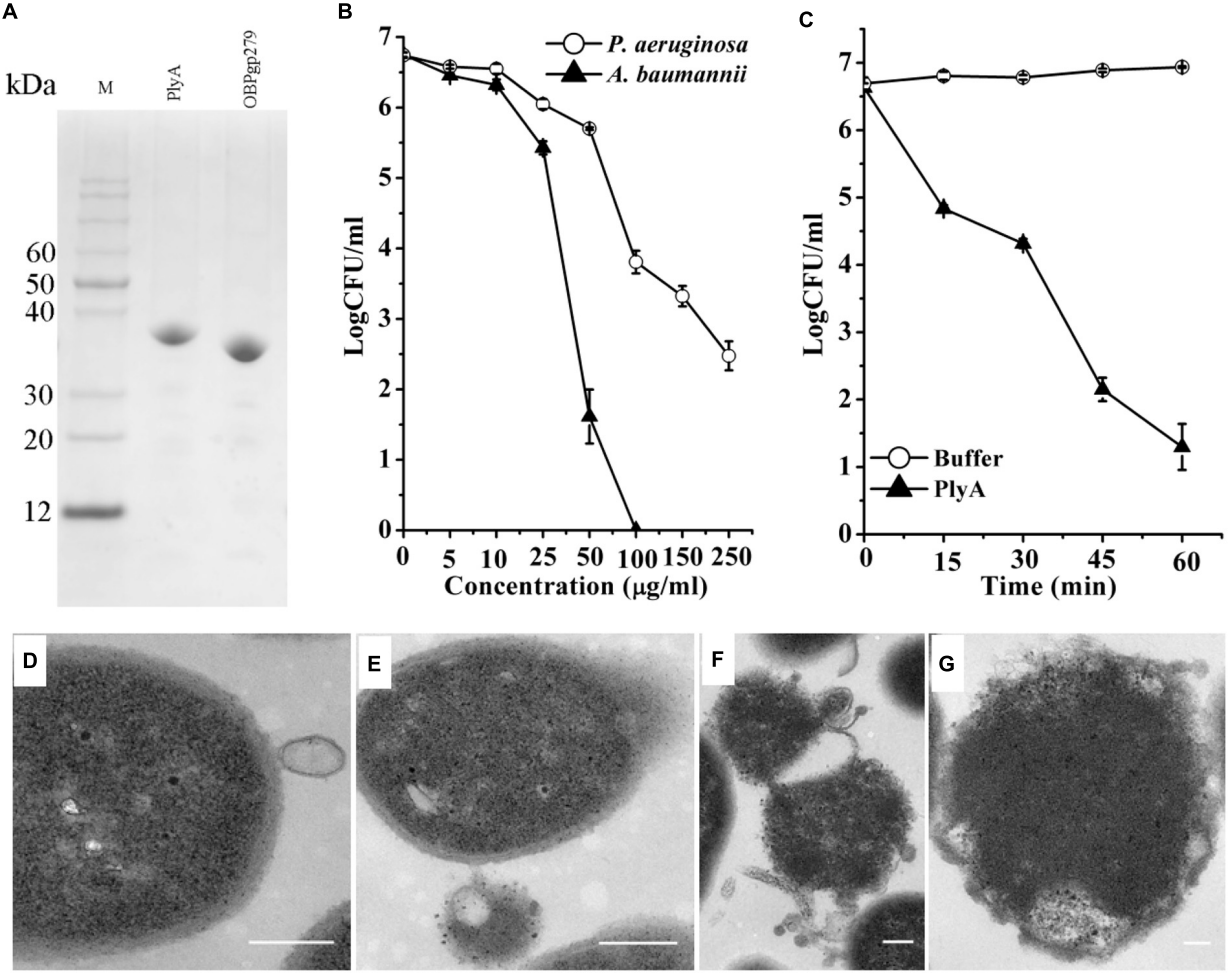
**Activity of peptide-modified lysin (PlyA) in Tris-HCl buffer. (A)** Analysis of purified proteins on 12% SDS-PAGE gel. **(B)** Dose-dependent antibacterial activity of PlyA against logarithmic *Acinetobacter baumannii* WHG3066 and *Pseudomonas aeruginosa* WHG3012 in 20 mM Tris-HCl (pH 7.4). **(C)** Time-killing curve of 50 μg/ml PlyA against logarithmic *A. baumannii* WHG3066. **(D–G)** Transmission electron microscope (TEM) images of logarithmic *A. baumannii* WHG3066 exposed to PlyA. TEM analysis revealed that *A. baumannii* cells exposed to 100 μg/ml PlyA suffered from extrusion **(D)** and rapid disruption at single **(E)** or multiple sites **(F)**, resulting in the loss of cytoplasmic contents and ultimately the formation of ghost cell **(G)**. Bar sizes: 200 nm.

As shown in **Figure 1A**, CA-fused OBPgp279 (called PlyA for short) and its parental lysin OBPgp279 were well expressed in *E. coli* as soluble proteins with purity of >95% as observed by 12% SDS-PAGE gel. Antibacterial activity tests showed that PlyA could kill logarithmic phase *A. baumannii* WHG3066 and *P. aeruginosa* WHG3012 cells rapidly in a dose-dependent manner (**Figure 1B**). Specifically, a reduction of over 5 logs (from 6.6 log to 1.2 log) was observed after treating the *A. baumannii* cells with 50 μg/ml PlyA for 1 h (**Figure 1B**), and a reduction about 2.5 logs (from 6.7 log to 3.8 log at 100 μg/ml PlyA) for *P. aeruginosa* cells (**Figure 1B**). Nearly no viable *A. baumannii* cells were detected when the concentration of PlyA increased to 100 μg/ml. While its parental lysin OBPgp279 (with equimolar) could only cause a reduction of about 1.38 logs (data not shown). The time kill curve revealed that the viable *A. baumannii* cell numbers were reduced about 1.8 logs (from 6.6 log to 4.8 log) within the first 15 min when treated with 50 μg/ml PlyA, and the killing continued for at least 60 min (**Figure 1C**).

Transmission electron microscope Images revealed that the *A. baumannii* WHG3066 cells exposed to PlyA suffered from leakage (**Figure 1D**) and rapid disruption at single (**Figure 1E**) or multiple sites (**Figure 1F**), resulting in the partial or total loss of cytoplasmic contents and ultimately loss of cell integrity (**Figure 1G**).

### Antibacterial Spectrum of PlyA

**FIGURE 2:**
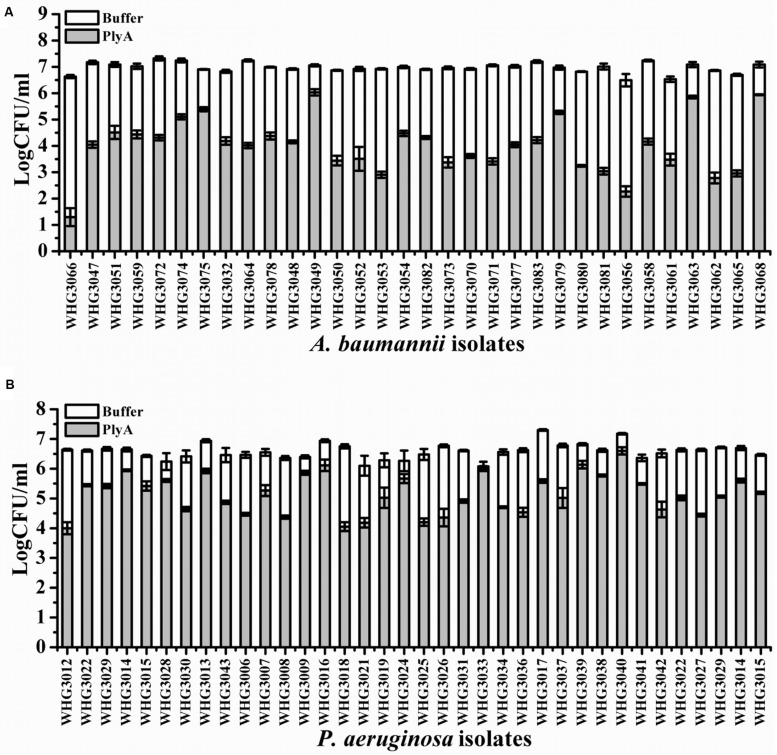
**Antibacterial activity of PlyA against clinical isolates of *A. baumannii* and *P. aeruginosa* in 20 mM Tris-HCl (pH 7.4). (A)** Antibacterial activity of PlyA (50 μg/ml) against *A. baumannii* clinical isolates in logarithmic phase at 37°C for 1 h. **(B)** Antibacterial activity of PlyA (100 μg/ml) against *P. aeruginosa* clinical isolates in logarithmic phase at 37°C for 1 h.

Next, we tested the susceptibility of a collection of clinical isolates of *A. baumannii* and *P. aeruginosa* to PlyA (in Tris-HCl), including another 31 *A. baumannii* isolates and 35 *P. aeruginosa* isolates with multiple antimicrobial resistances profiles (**Table 1**). The plating assay revealed that PlyA was active against all *A. baumannii* isolates tested, causing a reduction of 1.0–4.2 logs. Except WHG3033, all *P. aeruginosa* isolates tested (35/36) were susceptible to PlyA (with a reduction of 0.5–2.7 logs in viable cell number; **Figure 2**). The variable susceptibility of these clinical isolates to PlyA may be due to their different modifications in their outer membrane structure. No antibacterial activity of PlyA was observed against *Escherichia coli* BL21(DE3) and *Bacillus cereus* IS195 tested (data not shown). These results demonstrate that PlyA has broad antibacterial activity against MDR *A. baumannii* and *P. aeruginosa* isolates in Tris-HCl buffer.

### Bacterial Phase Affects the Antibacterial Activity of PlyA

**FIGURE 3:**
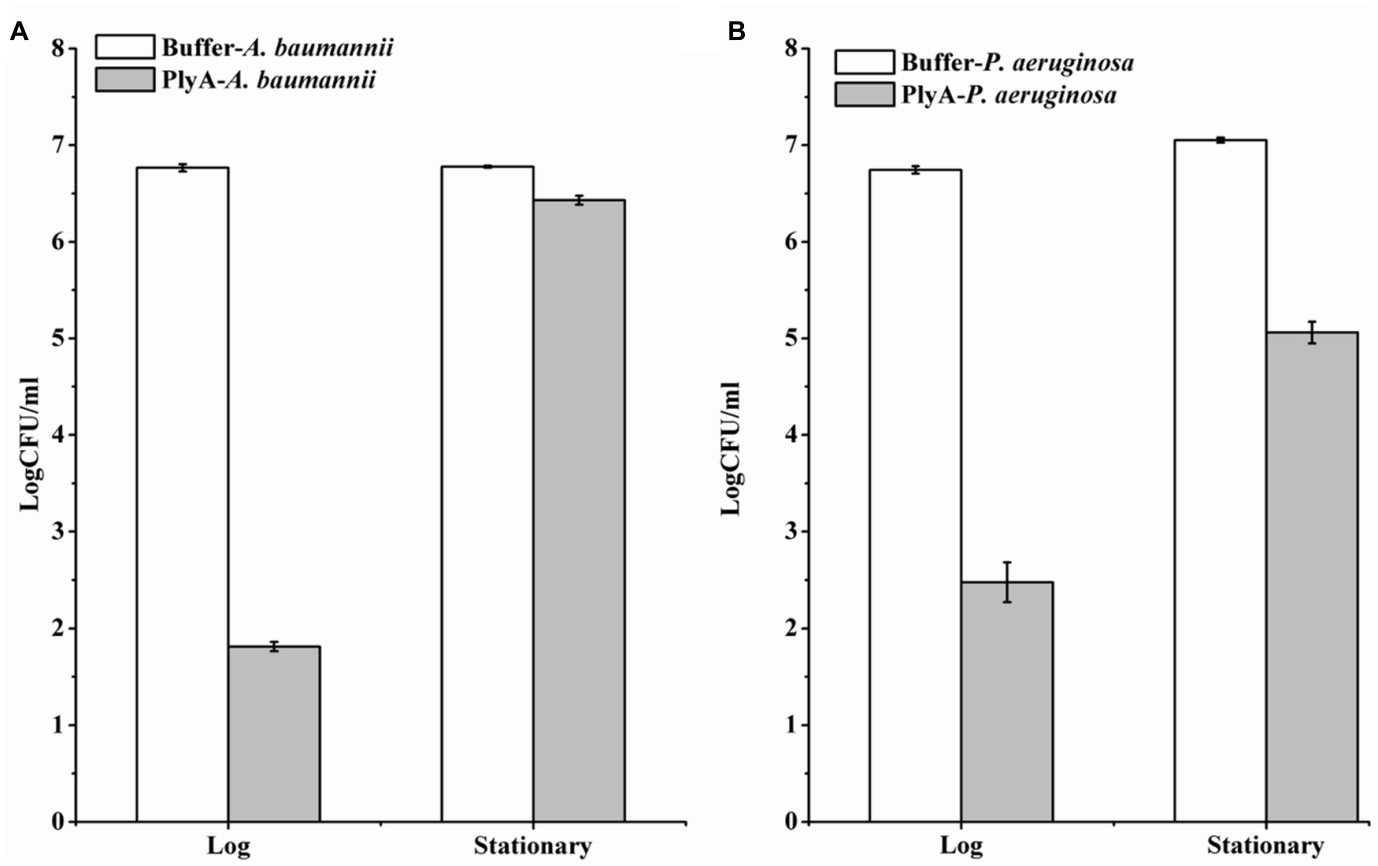
**Comparison between the activity of PlyA against *A. baumannii* WHG3066 **(A)** and *P. aeruginosa* WHG3012 **(B)** cells in logarithmic and stationary phase at 37°C for 1 h in 20 mM Tris-HCl (pH 7.4).** Concentration of PlyA: 50 μg/ml.

Although PlyA showed good antibacterial activity (from 6.8 log to 1.8 log) against logarithmic phase *A. baumannii* (**Figure 1C**), only a minor activity (from 6.8 log to 6.4 log) could be observed after treatment of the stationary phase *A. baumannii* WHG3066 cells with 50 μg/ml PlyA for 1 h (**Figure 3A**). Different susceptibilities to PlyA were also observed for logarithmic and stationary phase *P. aeruginosa* WHG3012 cells, but the stationary cells were still killed with a reduction of approximately 2 logs in cell number (**Figure 3B**). These results show that stationary phase *A. baumannii* and *P. aeruginosa* cells are not or partially killed in comparison to their logarithmic phase ones, respectively.

### OMPs Enhance the Antibacterial Activity of PlyA Against Stationary Phase Cells

**FIGURE 4:**
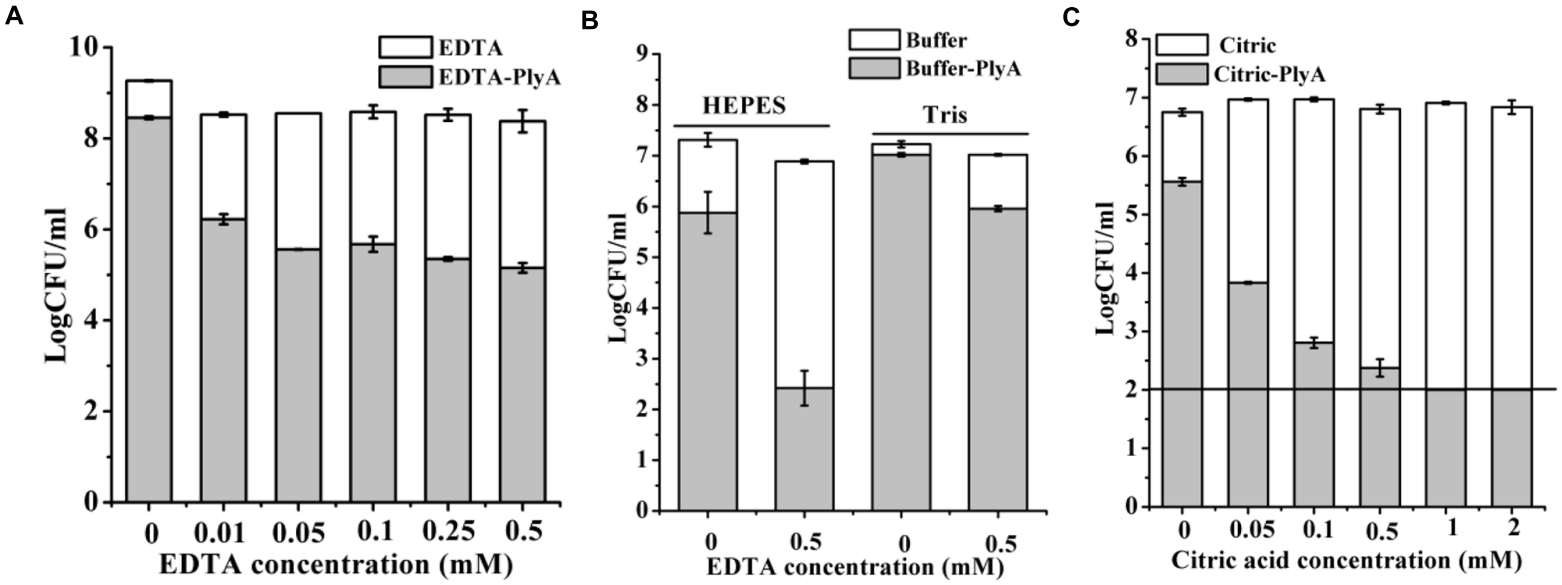
**Effects of EDTA and citric acid on the antibacterial activity of PlyA. (A,B)** Effects of 100 μg/ml PlyA combined with various concentrations of EDTA against stationary phase *A. baumannii* WHG3066 in 20 mM Tris-HCl (pH 7.4) **(A)**, and *P. aeruginosa* WHG3012 **(B)** cells in different buffers at 37°C for 1 h. **(C)** Effects of 100 μg/ml PlyA combined with various concentrations of citric acid against stationary phase *A. baumannii* WHG3066 in 20 mM Tris-HCl (pH 7.4) with pH adjustment.

Outer membrane permeabilizers (OMPs), such as EDTA and citric acid have been used to enhance the bacteriolytic activity of lysins (Oliveira et al., 2014). However, an unneglectable minor killing effect on logarithmic *P. aeruginosa* was noted in the presence of EDTA (Walmagh et al., 2012) and citric acid alone (Oliveira et al., 2014) previously by other researchers. Therefore, we only tested the synergism of these OMPs with PlyA against stationary phase cells. **Figure 4A** showed that EDTA alone has a minor killing effect on stationary phase *A. baumannii* cells, with a reduction of only 0.3 logs in 20 mM Tris-HCl (pH 7.4). Whilst, an obvious enhanced antibacterial activity of PlyA was observed in the presence of EDTA, with a reduction of 2.3–3.2 logs in the viable cell number. The synergism between EDTA and PlyA was observed not only in 20 mM Tris-HCl (pH 7.4), but also in 5 mM HEPES-NaOH (pH 7.4) against stationary phase *P. aeruginosa* (with a reduction of 4.4 logs; **Figure 4B**). Because the acidification effect of citric acid was reported to kill logarithmic cells (Oliveira et al., 2014; Briers and Lavigne, 2015), we tested the effect of citric acid (0.05–2 mM) on PlyA antibacterial activity against the stationary phase cultures of *A. baumannii* by adjusting the pH values of the mixtures to 7.4 after adding citric acid to the Tris-HCl buffer. Results showed that a citric acid dose-dependent synergism between citric acid and PlyA was observed (**Figure 4C**), indicating that mechanisms other than the acidification effect exist underlying the synergism between citric acid and PlyA against the stationary phase cells.

### Antibacterial Activity of PlyA in Bio-Matrix Conditions

**FIGURE 5:**
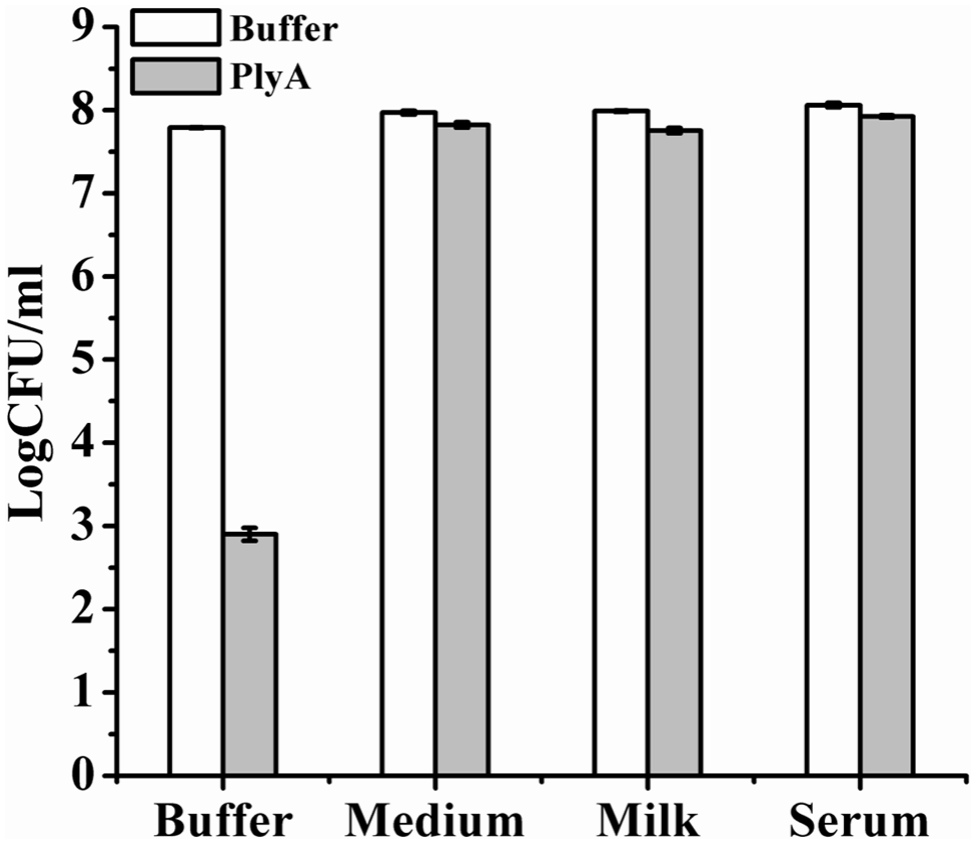
**Activity of PlyA in some matrices.** Logarithmic phase *A. baumannii* WHG3066 cells were suspended in Tris-HCl buffer (pH 7.4), LB culture medium (pH 7.0), pasteurized milk (pH 6.8), and serum (pH 6.9), respectively. After treatment with 100 μg/ml PlyA at 37°C for 1 h, the viable cell numbers were calculated by plating on LA plates. Buffer treated groups were used as controls.

Finally, we evaluated the performance of PlyA in some bio-matrix conditions, including LB culture medium, pasteurized milk, and human serum, which are more complicate than the pure Tris-HCl or HEPES-NaOH buffer. **Figure 5** showed that no antibacterial activity was observed in the presence of PlyA in these conditions against logarithmic phase *A. baumannii* cells. The abolished activity was also observed in MH, BHI, and TSBN culture media (data not shown). These results indicate that PlyA alone is easily inactivated in complicate environments, limiting its application only to relatively simple or special conditions.

## Discussion

The global emergence of MDR bacteria is calling to find novel molecules for treating the infections caused by them. Some recent studies on peptide-modified lysins or prophage lysins with similar structural compositions have shown that they could kill Gram-negative pathogens, including *P. aeruginosa* (Briers et al., 2014a) and *A. baumannii* (Lood et al., 2015). However, less is known about the conditions that influence the activities of peptide-modified lysins and their potential limitations in real applications. In the present study, we found that the activity of a new peptide-modified lysin could be affected by the bacterial growth phase and the bio-matrix, which should be taken into consideration for the development of new peptide-modified lysins.

We observed that PlyA showed good antibacterial activity in Tris-HCl buffer against logarithmic phase *A. baumannii* and *P. aeruginosa* clinical isolates (**Figure 2**), including these with various antibiotic resistance profiles (**Table 1**, some of them are MDR isolates). These results demonstrate that the strategy of modifying lysins with a selected peptide is powerful to obtain novel engineered lysins against Gram-negative bacteria, including MDR isolates. The action model of a well known peptide-modified lysin, Artilysin^®^, is speculated to be: (1) the peptide fused in the N- or C-terminal of a target lysin interacts with the lipopolysaccharide of the Gram-negative bacteria, resulting in the destabilization and deformation of the outer membrane; (2) the lysin moiety transfers through the outer membrane driven by the self-promoted uptake of the peptide, and (3) thus gets access to and hydrolyze its peptidoglycan substrates, ultimately gives rise to cell lysis. This hypothesized mode of action was recently confirmed by Briers and coworkers in the time-lapse microscopy of *P. aeruginosa* cells exposed to Artilysin^®^ LoGT-008 (Briers et al., 2014b). The TEM analysis of *A. baumannii* cells in this study also supports this hypothesis. As shown in **Figure 1**, PlyA disintegrates the cell wall of *A. baumannii*, and causes the loss of cytoplasmic contents in a single or multiple sites. This observation is quite similar with the typical phenomenon of osmotic-mediated cell lysis following the actions of phage lysins against Gram-positive bacteria reported elsewhere (Daniel et al., 2010).

Although PlyA showed good antibacterial activity against cells in logarithmic phase, a nearly abolished antibacterial activity was observed against stationary phase *A. baumannii* cells (**Figure 3A**). In case of stationary phase *P. aeruginosa* the antibacterial activity is not abolished but greatly reduced (**Figure 3B**). This observation is quite consistent with a recent report showing that a higher antibacterial activity of PlyF307 lysin was noted against exponentially growing *A. baumannii* (Lood et al., 2015). The different susceptibility may be due to the different structure and composition of the cell membranes between logarithmic and stationary phase cells. The outer membrane of bacteria is mainly composed of lipopolysaccharides, phospholipids and proteins, and their contents and types are varied in different environmental conditions and growth phases (Cronan, 1978). Additionally, there may be also difference in peptidoglycan architecture between logarithmic and stationary phase cells, such as the thickness of peptidoglycan layer, which influences their susceptibility to PlyA.

The strong synergisms between PlyA and OMPs (EDTA and citric acid in Tris-HCl and HEPES-NaOH buffer) against stationary phase cultures of *A. baumannii* (**Figures 4A,C**) and *P. aeruginosa* cells (**Figure 4B**) indicate that the outer membrane of Gram-negative bacteria is indeed a physical barrier for the bacteriolytic activity of natural lysins. By combining with OMPs, PlyA may be helpful in *ex vivo* and topical applications, such as environmental or surface disinfection, but not suitable for systemic infections due to the potential risk of anti-coagulating properties of the OMPs.

Other researchers have noted that protonated form of citric acid has both chelating effect and acidification effect on bacterial cells, and the acidification effect could damage the outer membrane of Gram-negative bacteria (such as *P. aeruginosa*) and cause a reduction in viable cell number (Oliveira et al., 2014; Briers and Lavigne, 2015). However, in the present study, we found that the acidification-killing effect of citric acid (2 mM) was only observed in logarithmic cultures of *A. baumannii*, but not stationary phase ones (data not shown). Moreover, an obvious synergism between citric acid and PlyA was observed against the stationary phase cultures of *A. baumannii* in a citric acid dose-dependent manner when pH values were adjusted to neutral pH (**Figure 4C**). These results indicate that it is the chelating effect, but not acidification effect of citric acid, which enhanced the antibacterial activity of PlyA against stationary phase cultures of *A. baumannii*.

One serious shortcoming for PlyA may be its inactivation in complicate bio-matrices, including media, milk and serum (**Figure 5**), which means PlyA could only be used in relatively simple or specified conditions, such as material and skin surface disinfection. One possible reason for the inactivation of PlyA may be the conjugation and passivation of negatively charged molecules present in these matrices to the positively charged peptides, which may render the peptide losing the outer membrane penetrating activity.

However, in the previous reports, the efficacies of some Artilysin^®^s have been demonstrated in *P. aeruginosa* infected *Caenorhabditis elegans* model and human keratinocytes monolayer model in the presence of EDTA (Briers et al., 2014b), as well as in *in vitro* case studies of dog otitis caused by *P. aeruginosa* (Briers and Lavigne, 2015). Therefore, it seems that it is not easy and straight-forward to design an engineered peptide-modified lysin which shows robust activity under infection conditions as described for Artilysin^®^s. Some optimization like fusing different peptides with different endolysins and combining with different linkers is required to obtain an antibacterial lysin with the desired properties and a robust activity (Briers et al., 2014b).

## Conclusion

We report here a newly engineered lysin, PlyA, with high bacteriolytic activity against *A. baumannii* and *P. aeruginosa in vitro*. This study also indicated that conditions such as bacterial growth phase and the bio-matrix can influence the antibacterial activity of PlyA, suggesting that there are still some limitations that should be taken into consideration for the development of new peptide-modified lysins, and optimization is needed to obtain an antibacterial lysin with robust antibacterial activity.

## Author Contributions

HY and HW conceived the study. HY and MW performed experiments. HY, JY, and HW analyzed data. HY and HW wrote the paper.

## Conflict of Interest Statement

The authors declare that the research was conducted in the absence of any commercial or financial relationships that could be construed as a potential conflict of interest.
